# Influence of science education on mental health of adolescents based on virtual reality

**DOI:** 10.3389/fpsyg.2022.895196

**Published:** 2022-09-29

**Authors:** Bo Wu, Changlong Zheng, Benjamin Huang

**Affiliations:** ^1^Faculty of Education, Northeast Normal University, Changchun, China; ^2^High School Attached to Northeast Normal University, Changchun, China; ^3^University of California, San Diego, San Diego, CA, United States

**Keywords:** VR, teenagers, mental health, influencing factors, treatment system

## Abstract

This work is developed to explore the form of mental health education, strengthen scientific educational ideas, and improve the effect of psychological therapy. Virtual reality (VR) technology is innovatively applied in adolescent mental health treatment and education. Based on this, the mental health treatment and system design based on virtual reality technology are discussed, and the feasibility of applying VR technology to adolescent mental health education is explored. Second, the research concept of adolescent mental health is discussed. Based on the VR platform setup, questionnaire survey is implemented to analyze the factors influencing the mental health of primary and secondary school students in Xi’an, Shaanxi Province (the permission of the adolescent guardian is obtained during the interview), and five factors are obtained. Based on this, the adolescent mental health treatment system based on VR is designed, and the effectiveness of the system is tested and evaluated. The results show that the integrated delay of the VR equipment used is 29 ms, which can effectively provide service. There are significant differences in mental health status among adolescents of different genders, different ages, only children and non-only children, parents’ accompaniment during growing up, and urban and rural adolescents. Finally, after 3 months of psychological treatment, the mental health score of the experimental group of teenagers is 50–55 points. However, the mental health scores of the control group remain at 56–65 points, indicating that the mental health treatment system designed in this work can effectively help the adolescents to improve their mental health, thus proving the effectiveness of the system. To sum up, this work provides scientific reference for adolescent mental health education in schools. Psychological treatment system can help teenagers improve their psychological problems and promote the development of mental health education.

## Introduction

This work is developed to explore the form of mental health education, strengthen scientific educational ideas, and improve the effect of psychological therapy. Virtual reality (VR) technology is innovatively applied in adolescent mental health treatment and education. Based on this, the mental health treatment and system design based on virtual reality technology are discussed, and the feasibility of applying VR technology to adolescent mental health education is explored. Second, the research concept of adolescent mental health is discussed. Based on the VR platform setup, questionnaire survey is implemented to analyze the factors that influence the mental health of primary and secondary school students in Xi’an, Shaanxi Province (the permission of the adolescent guardian is obtained during the interview), and five factors are obtained. Based on this, the adolescent mental health treatment system based on VR is designed, and the effectiveness of the system is tested and evaluated. The results show that the integrated delay of the VR equipment used is 29 ms, which can effectively provide service. There are significant differences in mental health status among adolescents of different genders, different ages, only children, and non-only children, parents’ accompaniment during growing up, and urban and rural adolescents. Finally, after 3 months of psychological treatment, the mental health score of the experimental group of teenagers is 50–55 points. However, the mental health scores of the control group remain at 56–65 points, indicating that the mental health treatment system designed in this work can effectively help adolescents to improve their mental health, thus proving the effectiveness of the system. To sum up, this work provides scientific references for adolescent mental health education in schools. Psychological treatment system can help teenagers improve their psychological problems and promote the development of mental health education.

With the development of society, education has become the main task of the current society, so it is very necessary to improve the learning ability of teenagers. To achieve, it is also necessary to improve the mental health of teenagers (Kingsbury et al., 2020). In other words, adolescent mental health education is an inevitable requirement of education modernization (Straatmann et al., 2019) and a difficult teaching task that schools should strive to do well. However, utilitarianism leads to frequent mental health problems among teenagers in the current education system. The exam-oriented education leads to the neglect the adolescents’ personality and a strong sense of psychological depression, which may lead to the occurrence of depression and other negative emotions. In addition, the concentration of mental health education in schools is not high, which is misunderstood by many people and lacks some formal and professional training (Urano et al., 2020). Moreover, even if mental health education is opened in schools, the traditional mental health education is mostly one-to-one or one-to-many dialog counseling, which has a single form, poor pertinence, and poor therapeutic effect. Therefore, the reform of adolescent mental health education is an indispensable part of education modernization reform, and it is urgent to innovate the way of adolescent mental health education (Finning et al., 2020; Pellegrini et al., 2020).

Virtual reality (VR) technology is adopted to provide technical support for teenagers’ mental health. The development of VR technology has brought new changes to human production and life. Its immersive experience can penetrate into and influence the user’s mental environment. Therefore, in the future education, the application of VR technology in adolescent mental health education will have a profound impact. How to further explore the application of VR technology in adolescent mental health education is the current mainstream. VR technology, as an advanced science and technology, can effectively provide users with an ideal perceptual environment through in-depth computer simulation of the real environment, so as to effectively improve the psychological status of users and play an important role in psychological therapy (Finan et al., 2020; Poap et al., 2020). VR technology provides ideal visual, auditory, tactile, and other guidance and adjustment for adolescents’ psychological therapy, which can enhance adolescents’ psychological adjustment ability and relieve psychological pressure.

Based on this, first, the factors that affect the mental health of teenagers are analyzed, and the VR technology is comprehensively analyzed. Then, VR technology is applied to adolescent mental health treatment and education, and based on this, the feasibility of VR technology applied to adolescent mental health education is analyzed. Finally, questionnaire survey is implemented to investigate the factors that affect the mental health of primary and middle school students in Xi’an city, Shaanxi Province, to test the effect of the system on psychological treatment. The innovation of this work lies in the investigation of adolescent mental health supported by VR technology based on psychological theory, a more comprehensive analysis of adolescent mental health problems, and the adolescent mental health treatment system designed by VR technology. This work not only provides scientific reference for adolescent mental health education and treatment, but also helps to perfect the current comprehensive adolescent education system.

## Related recent works

The application of VR technology in mental health treatment is a new research hotspot of traditional psychotherapy and mental health. VR technology restores the actual scene one-to-one and constructs the auditory, visual, and tactile fidelity scenes. For example, currently, music therapy and dance therapy shine in mental health treatment. The advantage of VR technology lies in the combination of the two and the integration of dance and music into the virtual scene. Therefore, VR technology for psychological therapy is the deepening of dance therapy and music therapy. For example, Hoover and Bostic (2021) pointed out that for most adolescents, the formation and development of intelligence development, moral cultivation, and improvement of comprehensive quality, as well as the formation and development of innovative consciousness, competitiveness, autonomous personality, and adaptability are affected by psychological quality. Therefore, it is a new task for educators to carry out adolescent mental health education scientifically and effectively. It is the basis and starting point of carrying out mental health education to analyze the present situation and cause of adolescent mental health and the situation and development trend of adolescent mental health. It is also the basis of adolescent psychological quality assessment. Scientific analysis and correct estimation of the degree of adolescent psychological disorders is an important premise of mental health education. Lin et al. (2020) proposed a VR system with the goal of reducing anxiety and stress, called virtual harmony music therapy. The system allows users to listen to music and play different instruments in a 3D environment with a real panoramic video as the background scene. It was verified whether the system can help to reduce stress and anxiety. The results showed that participants who enjoyed the music therapy system felt more relaxed and significantly less stressed. VR technology has strong adaptability and generalization ability and can construct scenes that meet patients’ psychological expectations according to the characteristics of different treatment objects. Farrell et al. (2020) applied VR technology to the treatment of children’s special phobias. The results showed that VR technology has a good clinical effect on the treatment of children’s special phobia. Pang (2019) designed a psychological relaxation system based on VR technology. The system can provide psychological relaxation for people with insomnia, mothers with postpartum depression, and college entrance examinees. VR psychological intervention relaxation system is more efficient than traditional relaxation. Zhang and Li (2021) divided 50 college students into the experimental group and the control group for psychological testing. The co-developed VR psychological relaxation training system was used to interfere with the experimental group, and the scores of the two groups were compared before and after the self-rating anxiety scale. The results showed that the mood of the experimental group improved significantly, while that of the control group did not. VR psychological relaxation training technology can be used as a competitive part of psychological intervention for college students.

From the above research, the application of VR technology in mental health treatment has no restriction on the treatment objects. In addition, VR technology can construct a virtual scene of exclusive psychotherapy according to the characteristics of patients. VR technology has many advantages in psychotherapy. VR system can be used to design a variety of environments close to reality. However, the current research results suggest that the application of VR technology in psychotherapy tends to be technical. Researchers are more inclined to construct perfect virtual scenes and tend to restore realistic scenes one-to-one, which is divorced from the goal of mental health treatment. As a result, although a good virtual scene was built, the therapeutic effect was not good. The reason lies in the poor pertinence of the virtual scene construction, and the mental health problems of the therapists were not fully evaluated before the virtual scene construction of psychological therapy. Although they built a relatively perfect virtual scene of mental health, the generalization ability was not enough. Hence, more research is needed to improve the VR technology in the aspect of mental health education. First, the hardware equipment of VR technology must be enhanced to improve the effect of VR technology in the application of psychological health education and improve the effect of psychological health education. The innovation of this work lies in that the adolescent mental health problems are fully understood with psychological theory as the basis and VR technology as the support to investigate the adolescent mental health based on the current works, and based on this, the adolescent mental health treatment system is designed *via* VR technology.

## Mental health therapy and system design based on virtual reality

### Characteristics and classification of virtual reality

**FIGURE 1:**
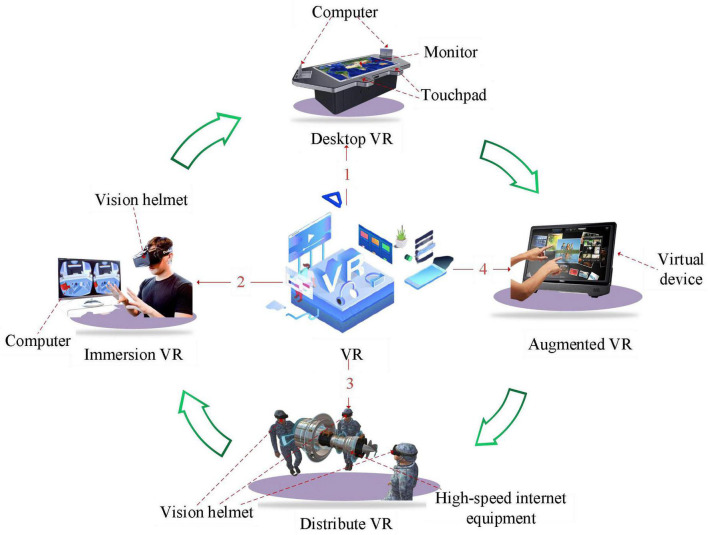
VR adoption scenarios.

With the rapid development of information technology, VR technology has been applied in all walks of life (Long et al., 2020). VR, as its name implies, is a comprehensive technology that makes use of multimedia graphics and image technology, sound effect, induction sensing, intelligent interface technology, psychology, and human–computer interaction to create a realistic virtual environment integrating sight, hearing, and touch (Hong and Kim, 2020; Sapkaroski et al., 2020). VR technology has three characteristics: immersion, interaction, and imagination (Abich et al., 2021; Reer et al., 2022). VR technology is classified into four types: (i) desktop (Guzsvinecz et al., 2021), (ii) immersive (Samosorn et al., 2020), (iii) distributed (Eiris et al., 2020), and (iv) augmented (Yu and Bowman, 2020). Figure 1 shows the fundamentals of the four types of VR technology.

### Basic theory of psychology and feasibility analysis of virtual reality

#### Basic theories of psychology

At present, adolescents are in the state of high intensity of learning. If adversely affected by the external environment, they may develop many psychological problems. One of the biggest effects on adolescents is depression, which is now the most common mental illness, with continuous and long-term depression as the main clinical characteristics. In addition, depression is the most important type of mental illness in modern people, especially adolescents. Clinically visible, the symptoms of depression are generally low mood for a long time to low depression, from the beginning of the unhappy to the final grief, inferiority, pain, pessimism, world-weariness, and finally even more suicidal tendencies and behavior. Patients often suffer from somatization symptoms, including chest tightness and shortness of breath. Depression is so difficult to detect, but it can greatly affect the modern society. Therefore, to protect the healthy growth of adolescents, it is necessary to effectively lower the impacts of psychological problems on adolescents’ lives through scientific education. In this work, the psychological status of adolescent students is tested effectively by answering questions in psychological science through questionnaire survey. The mental health grade of adolescents is graded through the result analysis, to intervene the psychological status of adolescents and help adolescents solve psychological problems.

#### Feasibility analysis of virtual reality technology in mental health education

The application of VR technology in mental health education is mainly the combination of VR technology and computer network technology (Showstark and Comshaw, 2020; D’Errico, 2021). The 3D realistic scene model is established, and various sensor devices and visualization devices are used to make young experiencers blend into the virtual environment from auditory, visual, and tactile aspects and get an immersive feeling. Teenagers can act as protagonists to truly feel the sense of the scene when the event happens (Abbas et al., 2020; Fransson et al., 2020). Based on this, VR technology is applied in the process of adolescent mental health education. By optimizing the hardware conditions of VR technology, the comprehensive effect of adolescent mental health education is improved. First, VR situations can break the limitations of time and space, and vivid and diverse scenes and events in the real society can inspire people to have rich and colorful emotional experience. However, in the practice of college students’ mental health education, due to the limitation of time, space, and other reasons, not everyone can have the opportunity to experience on site. VR technology enables young students to place themselves in a virtual environment to achieve psychological counseling, for example, VR training for fear of heights (Herrero and Lorenzo, 2020; Ponti et al., 2020). Second, VR technology can enhance students’ participation and experience. The traditional mental health education has poor pertinence and single form and cannot be applied to the case. It is difficult for a boring course to arouse students’ interest. The social environment of simulation can effectively visualize the real scene and make the scene more vivid and lifelike. In this way, it can meet the requirements of students’ interaction with the scene, making students change from a passive imager to an active experiencer, and enhance students’ degree of participation and control. Third, the introduction of virtual technology in mental health education can make psychological training intuitive and visual, so that young students can intuitively examine the cognitive process.

### Adolescent psychotherapy system based on virtual reality

#### Principle of mental health education based on virtual reality technology

Henrik Ehrsson, a famous British psychologist, used VR technology to induce “out-of-experience” and “self-consciousness” in subjects through experiments, which means that subjects can feel that they can escape from their “body,” “observing” themselves and their surroundings as a third person. The subjects felt that they had an out-of-body feeling (Scherz et al., 2020). The experimental process is as follows: (i) First, a camera is placed 2 m behind the subject to obtain the back posture, and then, the subject wears VR eyes, through which the images in the camera are visualized; (ii) Then, the testers swing a stick in front of the camera and touch the subject’s chest with the same motion. The subject describes his or her body as if it was in front of the camera; (iii) the subjects also responded when the researcher hits the camera with a hammer. The above shows that the subjects feel that their vision and touch are separated, making them feel a body that does not really exist (Zhu, 2020; Liu et al., 2021). To sum up, the use of VR technology for mental health education must follow the above basic principles, so as to give full play to the role of VR technology in mental health education. Based on this psychological principle, a VR psychotherapy system is designed.

#### Principle of mental health education based on virtual reality technology

**FIGURE 2:**
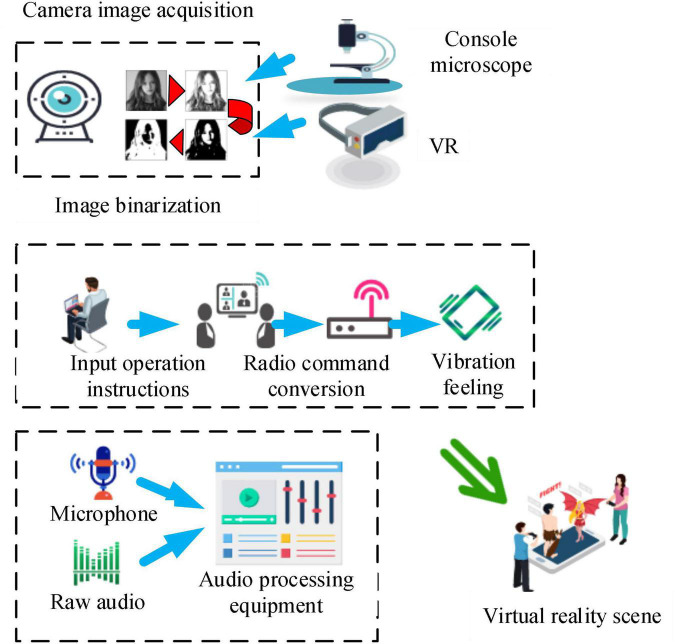
Structure chart of adolescent psychotherapy system.

Through the design of VR technology platform, psychological treatment system for teenagers is designed, and immersive VR design is adopted to build a virtual psychological platform for teenagers. The system requires an *in vitro* effect to distinguish between sight and touch in adolescent subjects. It increases the awareness of adolescents through verbal commands. Finally, the program should be flexible and able to make detailed changes according to the different psychological needs of teenagers. The adolescent psychotherapy system includes vision, touch, and hearing. The overall design is shown in Figure 2.

In Figure 2, the main camera of the visual part serves as the input device and is then transmitted to the image-processing unit *via* USB. The processing unit is responsible for the synthesis of the virtual environment, and the tactile part enhances the adolescent’s sense of immersion through the initial sensory receptors. In the auditory part, the microphone is used as input, and Dolby sound music is played according to the scene synthesized in the visual part and matched with the scene. As mentioned above, the use of VR technology must combine a series of basic skills of the human body, such as vision, hearing, and touch, so as to deeply intervene in the psychological changes in the human body and carry out a more comprehensive mental health education.

## Research on mental health of adolescents

With the progression of society, the mental health education of adolescents has become an important field in compulsory education. The age stage of adolescents is an important turning point in growing up, during which self-esteem, personality, even treason, and other psychological states often make them in a contradictory state of self-affirmation and self-denial (Desselle et al., 2020). Such negative psychological problems, if not timely dredge, will make adolescents in a negative mood, which will affect their healthy growth of adolescents. The important factors that affect the mental health of adolescents are investigated and analyzed, and a structural equation model is constructed to analyze the differences among different factors.

### Mental health of adolescents

**FIGURE 3:**
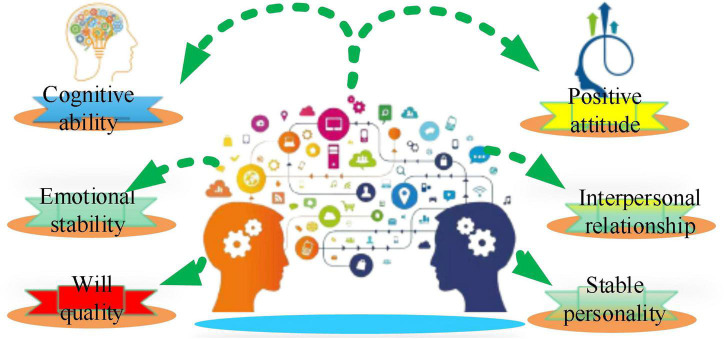
Standards for mental health of adolescents.

Through the way of the questionnaire survey, the psychological changes in students are assessed. The first is the student basic psychological interview, which refers to the analysis of the various factors that have an impact on the students. Then, the psychological health test is performed through the way of scoring comprehensive analysis of students’ mental health degree, to provide the reference for later experiment. Finally, virtual health education intervention is carried out. After the intervention, the psychological test is conducted to evaluate the effect of VR technology on adolescent mental health education. Furthermore, *Statistical Package for the Social Sciences* (SPSS) 22.0 is employed for quantitative analysis of adolescent mental health problems, and the influencing factors of adolescent mental health are obtained. Then, the mental health assessment model is constructed based on the psychology, sociology, and statistics, and *Analysis of Moment Structure* (AMOS) 22.0 is used to analyze the influencing factors. The mental health standards adopted to analyze adolescent mental health are shown in Figure 3.

Figure 3 demonstrates that there are six mental health standards for adolescents, namely, cognitive ability, emotional stability, will quality, positive attitude, harmonious interpersonal relationship, and stable personality. The specific analysis is as follows.

#### Construction of mental health index system

According to the standard, the mental health status of adolescents is analyzed, and “cognitive ability,” “emotional health,” “will quality,” and “positive attitude” are selected. The five factors that affect the mental health of adolescents are the partner effect, learning pressure, family background, family atmosphere, and school atmosphere (Table 1).

**TABLE 1:** Factors that affect the mental health of adolescents.

Influencing factors	Questionnaire questions
B school atmosphere	B1 You are often praised by the teacher (if there is, the effect is positive, if not, the effect is negative)
	B2 Your class has a good class style (if there is, the effect will be positive, if not, the effect will be negative)
	B3 In school life, consider yourself easy to get along with others (if there is, the effect will be positive, if not, the effect will be negative)
	B4 You feel close to people in this school (if there is, the effect will be positive, if not, the effect will be negative)
C family atmosphere	C1 How is your relationship with your parents (if there is, the effect will be positive, if not, the effect will be negative)
	C2 The relationship between parents is very good (if there is, the effect is positive, if not, the effect is negative)
	C3 How often do you and your parents do the following things together: reading, playing sports, visiting museums, zoos, science and technology museums, etc., going out to watch movies, performances, sports competitions, etc. (if there is, the effect is positive, if not, the effect is negative)
D family background	D1 The highest educational level of your parents
	D2 What is the current economic condition of your family?
G study pressure	G1 The pressure you feel about your parents’ expectations for your education is
	G2 You take extra-curricular tutoring classes: Math Olympiad, General Math (excluding Math Olympiad), Chinese/Composition, English (the more you attend, the greater the pressure)
	G3 The time you spent writing the homework assigned by the school teacher every day
H partner effect	H1 Does your good friend have any of the following situations: being criticized and punished for violating school discipline (if there is, the effect will be negative, if not, the effect will be positive)
	H2 Does your good friend have the following conditions: puppy love (if there is, the effect will be negative, if not, the effect will be positive)
	H3 Does your good friend have the following conditions: smoking, drinking (if there is, the effect will be negative, if not, the effect will be positive)

#### Data processing

The data of mental health of adolescents surveyed in this work come from the Database of China Education Panel Survey (CEPS). The database contains basic information such as household registration information, academic status, and family status of adolescents. Firstly, the data are screened according to the definition of adolescents (11–18 years old). Second, the original data are processed to facilitate the analysis and investigation. The continuous variables in the original questionnaire data are converted into sequential variables. The age of the respondents is calculated by subtracting the year of birth from the year of survey in the original survey data. The following is a standardized assignment of the factors that affect adolescent mental health. Regarding the original data, cognitive ability is graded into 5 intervals, with values of 1, 2, 3, 4, and 5, respectively. The will quality is reflected by the three questions: Q1: I can go to school even if I feel sick or something else. Q2: I will still try my best to do the homework I don’t like. Q3: Homework takes a lot of time and energy, and I will still stick to it. A five-level Likert scale is adopted, which is graded into “strongly disagree,” “disagree,” “generally,” “agree,” and “strongly agree.” One point is “strongly disagree” and five points is “strongly agree.” Emotional health is reflected by five aspects, namely, serious depression, depression, unhappiness, boredom, and sadness. A five-point scale graded into “always,” “often,” “sometimes,” “rarely,” and “never” is adopted. “Always” is 1 score and “never” is 5 scores. Positive attitude is reflected by a five-level scale graded into “not at all confident,” “not very confident,” “average,” “relatively confident,” and “very confident.” One score is “not at all confident” and five is “very confident.” From the above four steps, the scale for mental health of adolescents is fabricated.

#### Determining the evaluation index of influencing factors

##### School atmosphere

The school atmosphere is evaluated by four items according to Table 1, namely, “You are often praised by your teacher,” “The class you are in is nice,” “In school life, you think you are easy to get along with others,” and “You feel close to the people at this school.” A five-level Likert scale is adopted, which is graded into “strongly disagree,” “disagree,” “generally,” “agree,” and “strongly agree.” One point is “strongly disagree” and five points is “strongly agree.”

##### Family atmosphere

Family atmosphere is evaluated from four aspects according to Table 1. The frequency of parent–child interaction is utilized to analyze family atmosphere. Parent–child interaction content includes “reading,” “doing sports,” “visiting science museum,” and “watching movies, performances.” Likert scale of five levels of “never,” “once a year,” “once a half a year,” “once a month,” and “once a week” is adopted. One score is given for “never” and five for “once a week.”

##### Family background

According to Table 1, family background is divided into two dimensions of “parents’ highest level of education” and “family economic situation.” Parents’ highest level of education is divided into five grades, from primary school, junior high school, senior high school/technical secondary school, undergraduate/junior college, to postgraduate and above, with the order of 1–5 points. Family economic situation is also on a five-point scale, very difficult, relatively difficult, medium, relatively well off, and very well off, on a scale of 1–5.

##### Learning pressure

According to Table 1, learning pressure is divided into three dimensions. The pressure of parents’ expectation is 1–5 in descending order, the number of extracurricular remedial classes is 0–4, and the values are 5, 4, 3, 2, and 1, respectively. For 0–9 h to complete the homework every day, every 2 h is set as a level, and the values are assigned as 5, 4, 3, 2, and 1, respectively.

##### Partner effect

According to Table 1, the partner effect is divided into three dimensions. The scoring options are set as “many such,” “one or two such,” and “none such,” and assigned as 1, 3, and 5, respectively.

#### Model selection and establishment

Structural equation modeling (SEM) is a statistical method to analyze the relationship between variables based on the covariance matrix of variables, including factor model and structural model, reflecting the perfect combination of traditional path analysis and factor analysis. The analysis of structural equation model is roughly divided into four steps, namely, model construction, model fitting, model evaluation, and model modification. The structural equation model is adopted in this work to analyze adolescent mental health. The relationship between observed variables and latent variables of the measurement model of structural equation model can be written as the following equation.


(1) $$X=\wedge_{x}\,\xi +\delta$$

(2) $$Y=\wedge_{y}\,\eta +\varepsilon$$

where *X* is the vector composed of exogenous observation variables, *Y* is the vector composed of endogenous observed variables, _*δ*_ is the residual of *X*, and _*ε*_ is the residual of *Y*. The application effect of VR technology is verified through the latent variable and observation variable in the application process of VR technology.

The SEM equation measurement model must meet the following three conditions.


(3) E⁢(η)=0,E⁢(ξ)=0,E⁢(ε)=0,E⁢(δ)=0

(4) $$b^{0}\,\left(\varepsilon |\eta ,\xi ,\delta \right)$$

(5) $$b^{0}\,\left(\delta |\eta ,\xi ,\varepsilon \right)$$

where _*b^0^*_ is that variables are not correlated, so latent variables in SEM can be written as follows.


(6) η=B⁢η+Γ⁢ξ+ζ

where _*η*_ is the exogenous latent variable vector, _*ξ*_ is the endogenous latent variable vector, and _*ζ*_ represents the residual of the equation.

Then, the SEM model must meet the following two conditions.


(7) E⁢(η)=0,E⁢(ξ)=0,E⁢(ζ)=0

(8) $$b^{0}\,\left(\xi |\zeta \right)$$

To sum up, the latent variables in the proposed test model mainly include the influencing factors during the application of VR technology, whereas the observation variables mainly include the main influencing factors during the application of VR technology on the experimental objects. Since VR technology may cause certain operational errors in the application process, it is necessary to conduct a comprehensive analysis of these factors. The main research purpose was to analyze the impact of VR technology on experimental objects in the application process, so it is necessary to conduct a major study on these factors.

### Design of mental health therapy platform based on virtual reality

#### Design for system hardware and software

**FIGURE 4:**
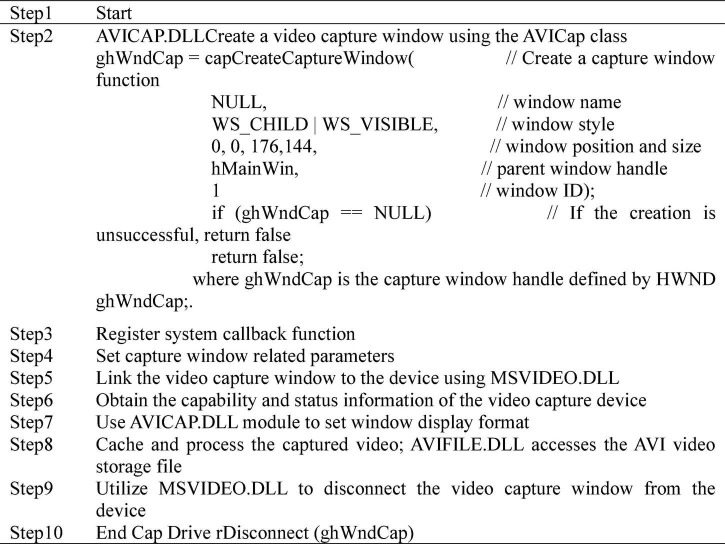
VFW video capture process.

Visual image acquisition is the most basic and key part of the whole system, and the quality of image acquisition determines the merits of the system. Digital video processing software progression package is utilized for video capture, and the biggest advantage of the software package is not too much dependence on the device, such as Video for Windows (VFW) and DirectShow digital Video processing software package. VFW is installed in Windows, and the installation program automatically installs video components required for video configuration, such as device drivers and video compression programs. The main advantage of VFW image acquisition is that it is equipped with a simple and easy to operate driver requiring no special hardware support, which can quickly complete video capture and single frame video acquisition, and can directly access the video buffer without producing intermediate files. Therefore, VFW is utilized as the video capture software package, and DS-2CD3320D camera and Ethernet interface are employed as the acquisition device. The VFW consists of six parts of dynamically linked library AVICAP.DLL, MSVIDEO.DLL, MCIAVI.DRV, AVIFILE.DLL, Installable Compression Manager (ICM), and Audio Compression Manager (ACM). AVICAP.DLL performs video capture. MSVIDEO.DLL links the video capture window with the device, which can carry out ICM video coding service. MCIAVI.DRV plays back the captured video. AVIFILE.DLL supports access to AVI files. ICM compresses and decompresses the captured video. ACM provides a similar service to ICM, except that it applies to wavy audio. AVICap is the main medium for video operation. The AVICAP.DLL module is utilized to create a video capture window, which mainly completes the following tasks: (i) audio and video collection and storage in AVI files; (ii) control of the connection and disconnection between video and audio acquisition Windows and equipment; (iii) support for previewing and overlaying two display modes; (iv) control of the number of frames of video collection; (v) selection of video source, video format, and video compression mode; and (vi) save of the single frame video device-independent bitmap (DIB). The video capture process using VFW is shown in Figure 4.

**FIGURE 5:**
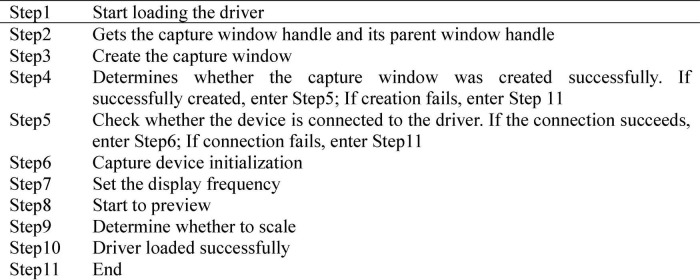
VFW driver loading process.

According to the system requirements, DS-2CD3320D video capture device is transmitted through Ethernet signal. The main process of loading VFW driver is shown in Figure 5. Camera driver process includes capture window creation, device driver connection, and initialization settings in turn. If either of the first two steps in the process fails, the driver fails to load. The parent window of capture window is graphic control, and the display frequency is set to 60 Hz.

#### Human target recognition

Image target recognition is an important field in artificial intelligence. Regarding the characteristics of adolescent psychotherapy system, human target recognition must be fast, accurate, and real-time tracking. Therefore, the adaptive Gaussian background difference method is utilized to detect the target in this study. The background difference method is utilized to detect the human body, and the difference image between the current frame and the background is expressed in Equation (9).


(9) $$D_{K}\,\left(x,y\right)=||F_{K}\,\left(x,y\right)-B_{K}\,\left(x,y\right)||$$

In the above equation, _(x,y)_ is the coordinate position of pixel points, _*F_K_ (x,y)*_ is the current frame image of the video sequence, *k* is the first frame of the video sequence, and _*B_K_ (x,y)*_ is the current background image.

The pixel points are determined according to the characteristics of human target foreground image and background image, as presented in Equation (10).


(10) $$R_{K}\,\left(x,y\right)=\left\{ \begin{array}{c} 1,D_{K}\,\left(x,y\right)≻T\\ 0,D_{K}\,\left(x,y\right)\leq T \end{array}\right.$$

In the above equation, *T* is the appropriate threshold for segmentation background and foreground in target detection.

Establishing an appropriate background model to reflect the background changes in time is necessary, so that the background image can accurately reflect the background of the current frame. Mixed Gaussian model is a multi-background model that can adapt to multi-background targets. Moving target detection using mixed Gaussian model is roughly summarized as the following steps.

First, the Gaussian mixture model (GMM) probability distribution density function is expressed as Equations (11)–(13).


(11) $$P\,\left(x_{i}\right)=\sum_{j=1}^{m}w_{j}\,g_{j}\,\left(x_{i};\mu_{j};\sigma_{j}\right)$$

(12) $$\sum_{j=1}^{m}w_{j}=1$$

(13) $$g\,\left(x_{i};\mu_{j},\sigma_{j}\right)=\frac{1}{{\left(2\,\pi \right)}^{\frac{1}{2}}\,{\left|\sigma_{j}\right|}^{\frac{1}{2}}}\,e^{-\frac{1}{2}\,{\left(x-\mu_{j}\right)}^{T}\,\sigma_{j}^{-1}\,\left(x-\mu_{j}\right)}$$

In the above equations, _*g(x_i_;μ _j_,σ _j_)*_ is the probability density function of the j-th single Gaussian model. To simplify the expression method, the function composed of three parameters, namely, weight _*w_j_*_, mean _*μ_j_*_, and covariance _*σ_j_*_, is represented by Equation (14).


(14) $$\Psi_{j}=\left(w_{j},\mu_{j},\sigma_{j}\right)$$

The GMM is composed of *M* single Gaussian model. All parameters of the GMM are estimated through the calculation of the sample set *X*, as presented in Equation (15).


(15) $$\Phi ={\left(\Psi_{1},\Psi_{2},\ldots ,\Psi_{M}\right)}^{T}$$

The probability equation for sample point _*x_i_*_ is shown in Equation (16).


(16) $$p\,\left(x_{i}|\Phi \right)=\prod_{i=1}^{N}\sum_{j=1}^{M}w_{j}\,g_{j}\,\left(x_{i};\mu_{j};\sigma_{j}\right)$$

In the above equation, *N* is the number of data, _*w_j_*_ is the proportion of the j-th single Gaussian model in GMM, and _*gj(x_i;_ μ_j_; σ_j_)*_ is the probability density function of the j-th single Gaussian model.

Then, the expectation maximization (EM) algorithm is employed to estimate the parameters of the GMM. The EM algorithm can optimize the data, thereby deepening the practicality of the research results and providing an important reference for the validation of the model. In addition, EM algorithm can accurately analyze the overall psychological status of the current adolescents and improve the reliability of the research content. For the convenience of calculation, if Equation (11) is assumed, Equation (11) is converted to Equations (17) and (18).


(17) $$p\,\left(x_{i}\right)=w_{1}\,g\,\left(x_{i};\mu_{1},\sigma_{1}\right)+w_{2}\,g\,\left(x_{i};\mu_{2},\sigma_{2}\right)+w_{3}\,g\,\left(x_{i};\mu_{3},\sigma_{3}\right)$$

(18) $$w_{1}+w_{2}+w_{3}=1$$

$\sigma_{j}^{2}\,I$ represents the covariance, where *I* represents the multidimensional identity matrix, and the covariance equation is shown in Equation (19).


(19) $$\sigma_{j}=\sigma_{j}^{2}\,I=\sigma_{j}^{2}\,\left[\begin{array}{cccc} 1 & 0 & \ldots & 0\\ 0 & \ldots & \ldots & \ldots \\ \ldots & \ldots & \ldots & 0\\ 0 & \ldots & 0 & 1 \end{array}\right],j=1,2,3$$

At this point, the single Gaussian density function is expressed by Equation (20).


(20) $$g\,\left(x_{i};\mu ,\sigma^{2}\right)={\left(2\,\pi \right)}^{-\frac{d}{2}}\,\sigma^{-d}\,e^{-\frac{{\left(x_{i}-\mu \right)}^{T}\,\left(x_{i}-\mu \right)}{2\,\sigma^{2}}}$$

In the above equation, *d* is the dimensional space. Then, Equation (17) is simplified into Equation (21).


(21) $$p\,\left(x_{i}\right)=w_{1}\,g\,\left(x_{i};\mu_{1},\sigma_{1}^{2}\right)+w_{2}\,g\,\left(x_{i};\mu_{2},\sigma_{2}^{2}\right)+w_{3}\,g\,\left(x_{i};\mu_{3},\sigma_{3}^{2}\right)$$

To obtain the optimal values of *μσw*, the optimal probability estimation algorithm is utilized to obtain the minimum value, as presented in Equation (22).


(22) $$J\left(\mu ,\sigma ,w\right)=\ln\left[\prod_{i=1}^{n}p\left(x_{i}\right)\right]=\sum_{i=1}^{n}\ln[w_{1}g\left(x_{i},\mu_{2},\sigma_{1}^{2}\right)+$$

$$w_{2}g\left(x_{i},\mu_{2},\sigma_{2}^{2}\right)+w_{3}g\left(x_{i},\mu_{3},\sigma_{3}^{2}\right)]$$

The process of the EM algorithm is as follows.

The first step is parameter initialization. It is assumed that *σ*_*j*_ is the unit covariance matrix, *μ*_*j*_ is a random number, and the weight occupied by each single Gaussian model in the mixed model is again shown in Equation (23).


(23) $$w_{j}=1\,/\,M$$

The second step refers to the estimation of the posterior probability of each sample. The values of initialization parameters obtained in the first step are utilized to calculate the posterior probability, as presented in Equation (24).


(24) $$\beta_{i\,j}=\frac{w_{j}\,g_{j}\,\left(x_{i};\Phi \right)}{\sum_{k=1}^{m}w_{k}\,g_{k}\,\left(x_{i};\Phi \right)}$$

1≤i≤n,1≤j≤m.

The third step is the maximization. To find the optimal weight of the mean, variance matrix, and single Gaussian model in GMM, the function *J*(*μ,σ*,*w*) takes partial derivatives of the above three parameters and requires reciprocal to be 0, so the updated weight is obtained. The mean and variance matrix are shown in Equations (25)–(27).


(25) $$w_{j}=\frac{\sum_{i=1}^{n}\beta_{i\,j}}{n}$$

(26) $$\mu_{j}=\frac{\sum_{i=1}^{n}x_{i}\,\beta_{i\,j}}{\sum_{i=1}^{n}\beta_{i\,j}}$$

(27) $$\sigma_{j}=\frac{\sum_{i=1}^{n}\beta_{i\,j}\,\left(x_{i}-\mu_{j}^{T}\right)\,{\left(x_{i}-\mu_{j}^{T}\right)}^{T}}{\sum_{i=1}^{n}\beta_{i\,j}}$$

The fourth step is the end-of-loop condition, which should be iterated to update the parameters until the function satisfies Equation (28).


(28) $$|p\left(x_{i}|\Phi \right)-p\left({\hat{x}}_{i}|\Phi \right)|≺\varepsilon$$

#### Video synthesis

The binarization of foreground and background is realized by Gaussian background difference. The result obtained is a binary image. The area and boundary coordinates of each foreground area are recorded during binary image marking. When the following two equations are met, it is judged as human body region.


(29) $${\text{A}}_{\min}<A<{\text{A}}_{\max}$$

(30) $$\text{bp}<\frac{y_{\max}-y_{\min}}{x_{\max}-x_{\min}}$$

In the equations above, *A* is the area of binary image area, *A*_*min*_ is the lower limit of recognition ability, *A*_*max*_ is the upper limit of recognition ability, *bp* is the proportional limit of height ratio and body width ratio, and *x,y* are the horizontal and vertical coordinates of boundary points.

**FIGURE 6:**
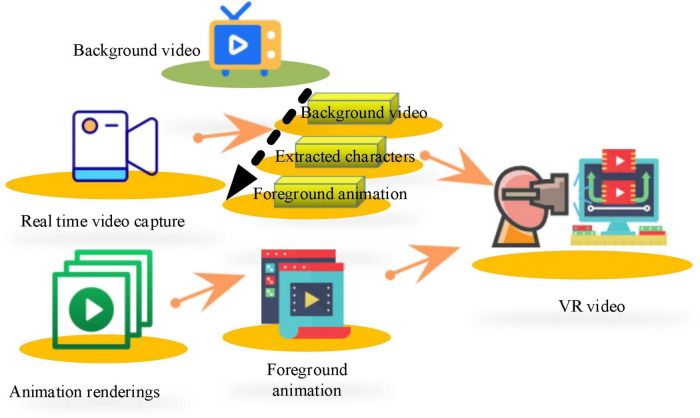
Video composition diagram.

After the video is collected, the second unit of image processing is utilized to synthesize the video, which is related to the immersion of the subjects in the process of psychological therapy. The video image synthesis diagram is shown in Figure 6.

**FIGURE 7:**
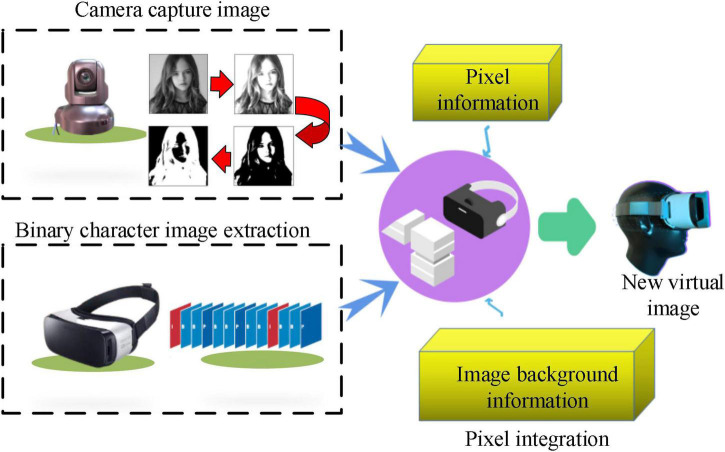
Image synthesis process.

The background video, real-time portrait, and foreground animation are superimposed in turn to generate a synthetic video. There are two fundamental reasons to transform color image into binary image processing. One is to reduce the data processing capacity of the first processing unit, and the other is to replace pixels accurately and quickly with unique binary image information during image synthesis of the second processing unit. The image synthesis process is shown in Figure 7.

Giving that there is a sense of layering in the images synthesized by real-time superposition that seriously affects the authenticity of the virtual scene, it is necessary to smooth the synthesized images. Median filtering is utilized for image smoothing, as mentioned above.

#### Experimental design of psychotherapy system

According to the results of the adolescent psychological survey, 40 adolescents with mental health problems are recruited and divided into an experimental group and a control group. The implementation settings are as follows.

In the experimental group, there are 20 people (10 boys and 10 girls), and the designed mental health treatment system is adopted for a 3-month psychological treatment. A professional psychologist is hired to consult 20 adolescent subjects every 10 days. The psychological state of the subjects is scored and recorded according to the Chinese Adolescent Mental Health Diagnostic Test (MHT) revised by Chinese psychologist Zhou Bucheng. The MHT has been widely used in more than 20 provinces and cities in China and shows high reliability and validity. The split reliability ranges from 0.85 to 0.88, and the test–retest reliability ranges from 0.667 to 0.863. The contents of the scale include learning anxiety (LA), social phobia (SP), loneliness tendency (LT), self-accusation (SA), sensitive tendency (ST), physical symptoms (PS), terrorist tendency (TT), and impulsive tendency (IT). The scoring system for this scale is as follows. There are three levels of mental health. Then, 1–55 points are normal, 56–64 points are poor or have problems, and more than 65 points are serious. In addition, specialized psychologists are hired to conduct psychological tests on the subjects every 10 days and rate them.

In the control group, there are 20 people (10 boys and 10 girls). They do not receive mental health treatment, and a specialized psychologist is hired to conduct psychological tests and score every 10 days.

A questionnaire survey is used to investigate the psychological status of young students. The main research content of the questionnaire is to investigate the psychological state changes in adolescent students of different genders before and after the experimental intervention to a certain extent and to evaluate the effect according to the research results. The reliability and validity tests show that Cronbach’s alpha coefficient of the design questionnaire content is 0.85, and the Kaiser–Meyer–Olkin (KMO) test coefficient is 0.796, which is higher than 0.5. The significant probability P of the Bartlett ball test is 0.002, which is lower than 0.05, and the questionnaire showed good reliability and validity. Based on the above research, it can fully compare the psychological changes in young people under the VR education system and the traditional education system, so as to fully reflect the role of the VR education system.

## Mental health of adolescents and virtual reality health system assessment

### Performance evaluation of virtual reality

**FIGURE 8:**
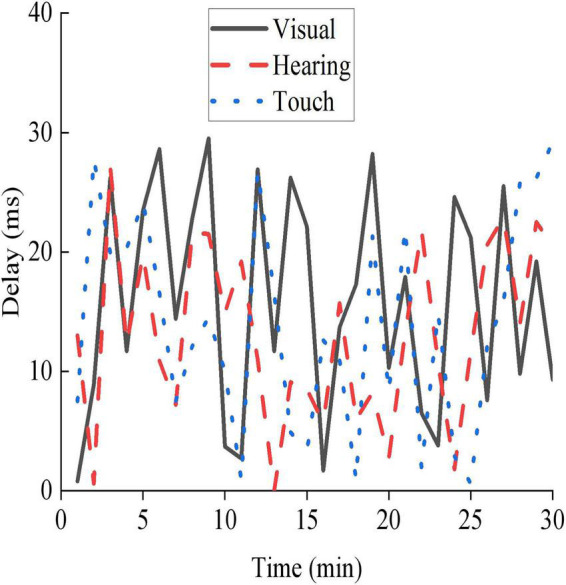
Basic performance testing status of VR equipment.

Virtual reality technology is adopted to intervene the psychological condition of adolescents, thereby comprehensively improving the quality of the current education of adolescents. Figure 8 shows a basic performance test of the VR appliance used in this work.

In Figure 8, the maximum delays of the VR equipment in visual perception, auditory perception, and tactile perception are 29, 27, and 29 ms, respectively. The VR equipment adopted shows strong practicability and can effectively provide VR services for users.

### Mental health of adolescents

**FIGURE 9:**
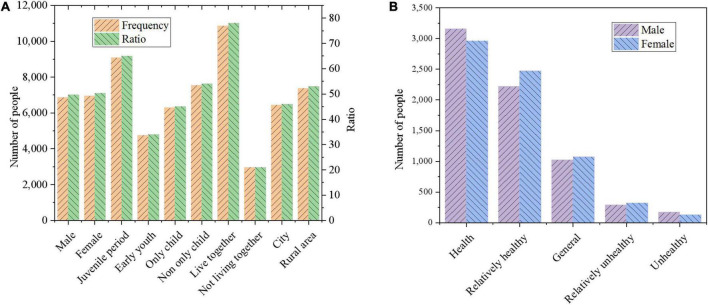
Comparison of mental health status of adolescents with different genders. **(A)** Demographic information; **(B)** mental health status of adolescents by gender.

The data utilized in this work are from CEPS, taking 438 classes in 112 primary and secondary schools in Changchun, Jilin Province, as the survey objects. First, demographic information and mental health status of adolescents of different genders are collected, as presented in Figure 9.

Figure 9A shows the basic composition of the adolescents investigated in this work, based on which the number of adolescents in different categories and their proportions in the overall population are clearly observed. The proportions of boys and girls are almost similar. Therefore, it is very important to compare various psychological conditions between the two when the ratio of boys and girls is the same. As illustrated in Figure 9B, in the two dimensions of “healthy” and “relatively healthy,” the proportion of boys is 78.29%, and the proportion of girls is 78.09%. In the two dimensions of “unhealthy” and “relatively unhealthy,” the proportion of boys is 6.78%, and the proportion of girls is 6.53%, and there is less difference in the psychological state of boys and girls. In addition, the mental health of boys is polarized and more stable than girls. The reason is that girls develop earlier and mature psychologically faster than boys. In addition, boys may be more rebellious and unwilling to communicate with others, resulting in more prominent psychological problems. Students’ psychological status is graded according to different ages, only-child and non-only-child, growth environment, and family situation. First, the mental health status of adolescents of different age groups is surveyed and statistically analyzed. People with 11–14 years old are in the juvenile period, whereas those with 15–18 years old are in the early youth period. The results are shown in Table 2.

**TABLE 2:** Health status of adolescents in different age groups.

	Juvenile period (People)	The early youth (People)	Total (People)
Healthy (People)	4,318 (47.52–70.51%)	1,806 (38.01–29.49%)	6,124 (44.26–100%)
Relatively healthy (People)	3,014 (33.17–64.24%)	1,678 (35.32–35.76%)	4,692 (33.91–100%)
Generally healthy (People)	1,213 (13.35–57.76%)	887 (18.67–42.24%)	2,100 (15.17–100%)
Relatively unhealthy (People)	357 (3.83–57.77%)	261 (5.49–42.23%)	618 (4.47–100%)
Unhealthy (People)	184 (2.04–60.73)	119 (2.51–39.27%)	303 (2.19–100%)
Total (People)	9,086 (100–65.66%)	4,751 (100–34.34%)	13,837 (100%)

The mental health problems of adolescents occur during the early youth. The “healthy” adolescents are 9.51% more than the early youth, and the “unhealthy” grade of adolescents is lower than that of the early youth. From the “unhealthy” and “relatively unhealthy” dimensions, the proportion of early youth is larger than that of adolescence. The reason is that young people face academic pressure and related psychological burden. Then, the psychological health of children from multiple families and children from only one family is statistically analyzed, and the results are shown in Table 3.

**TABLE 3:** Mental health status of adolescents from families with multiple children and families with only one child.

	Only-child (People)	Non-only-child (People)	Total (People)
Healthy (People)	3,013 (47.92–49.19%)	3,112 (41.19–50.81%)	6,125 (44.25–100%)
Relatively healthy (People)	1,920 (30.54–40.91%)	2,773 (36.72–59.09%)	4,693 (33.91–100%)
Generally healthy (People)	916 (14.57–43.59%)	1,185 (15.69–56.41%)	2,101 (15.18–100%)
Relatively unhealthy (People)	275 (4.38–44.43%)	344 (4.55–55.57%)	619 (4.47–100%)
Unhealthy (People)	163 (2.59–53.79%)	140 (1.85–46.21%)	303 (2.19–100%)
Total (People)	6,287 (100–45.42%)	7,554 (100–54.58%)	13,841 (100%)

Table 3 shows that the proportion of non-only-child in both “healthy” and “unhealthy” grades is lower than that of only-child, indicating that there are differences in the mental health of only-child adolescents. In the dimension of “healthy” and “unhealthy,” the proportion of only-child is 77.32%, while that of non-only-child is 77.28%. In the dimension of “unhealthy” and “relatively unhealthy,” the proportion of only-child is 7.41%, and that of non-only-child is 6.71%. The mental health of non-only-child is better than that of only-child. The reason may be that the only family pampers the adolescents too much, leading to poor psychological tolerance. Then, a survey is conducted on the mental health of adolescents with or without their parents in their growing up. The results are shown in Table 4.

**TABLE 4:** Mental health status of adolescents growing up with or without parents.

	With parents	Without parents	Total
Healthy (People)	4,998 (46.04–81.63%)	1,125 (37.75–18.37%)	6,123 (44.26–100%)
Relatively healthy (People)	3,630 (33.44–77.42%)	1,059 (35.54–22.58%)	4,689 (33.89–100%)
Generally healthy (People)	1,565 (14.42–74.52%)	535 (17.95–25.48%)	2,100 (15.18–100%)
Relatively unhealthy (People)	442 (4.07–71.52%)	176 (5.91–28.48%)	618 (4.47–100%)
Unhealthy (People)	220 (2.03–72.13%)	85 (2.85–27.87%)	305 (2.20–100%)
Total (People)	10,855 (100–78.46%)	2,980 (100–21.54%)	13,835 (100%)

From Table 4, in the “health” grade, the proportion of adolescents with only one parent or both parents at home is 37.9%, which is significantly lower than the proportion of adolescents with both parents at home is 45.99%. The percentage of adolescents with only one or both parents at home is significantly higher on the “unhealthy” and “relatively unhealthy” scales than teens with both parents at home. Teens with both parents at home report significantly better mental health than teens without parents. Mental health of adolescents and parental companionship are crucial. Then, the mental health of urban and rural adolescents is compared, and the results are shown in Table 5.

**TABLE 5:** Mental health status of adolescents in urban and rural areas.

	Urban (People)	Rural (People)	Total (People)
Healthy (People)	3,006 (46.63–49.09%)	3,118 (42.19–50.91%)	6,124 (44.26–100%)
Relatively healthy (People)	2,020 (31.33–43.05%)	2,672 (36.16–56.95)	4,692 (33.91–100%)
Generally healthy (People)	955 (14.81–45.48%)	1,145 (15.49–54.52%)	2,100 (15.18–100%)
Relatively unhealthy (People)	293 (4.54–47.41%)	325 (4.40–52.59%)	618 (4.46–100%)
Unhealthy (People)	173 (2.68–57.09%)	130 (1.76–42.91%)	303 (2.19–100%)
Total (People)	6,447 (100–46.59%)	7,390 (100–53.41%)	13,837 (100%)

As shown in Table 5, in terms of “unhealthy” grade, the proportion of urban adolescents is 2.71%, which is significantly higher than that of rural adolescents. In terms of “relatively healthy” grade, the proportion of rural adolescents is significantly higher than that of urban adolescents. There are fewer rural adolescents on the “less healthy” scale than urban ones. In general, the differences in mental health of rural adolescents are not obvious, and most of them are healthy psychological states. The fast pace of urban schools leads to greater pressure than that of rural students.

### Statistical inference analysis of influencing factors for mental health of adolescents

**FIGURE 10:**
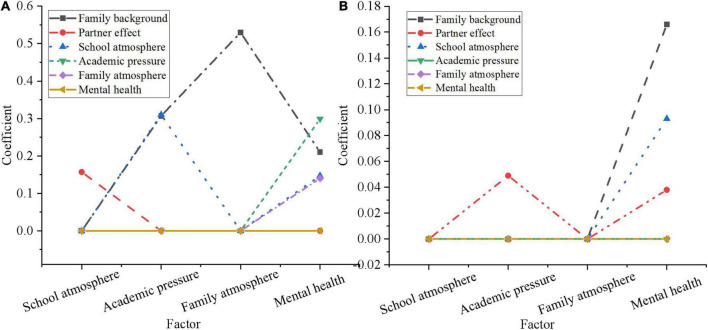
Statistical inference analysis of influencing factors of mental health of adolescents. **(A)** Standardized direct utility; **(B)** standardized indirect utility.

Structural equation modeling is utilized to analyze the factors that affect mental health through the normalization of direct utility and standardization of indirect utility. The results are shown in Figure 10.

According to Figure 10, “learning pressure,” “family background,” “school atmosphere,” and “family atmosphere,” all have significant impacts on adolescents’ mental health, among which “learning pressure” is the most significant with a coefficient of 0.299. This also shows that the falser pressure on adolescents, the more adverse to their mental health progression. The standardized coefficient of “family background” is 0.210, indicating that adolescents from families with high educational level and good economic situation are psychologically healthy. The coefficients of “school atmosphere” and “family atmosphere” are 0.147 and 0.140, respectively, indicating that a good environmental atmosphere plays a certain positive role in the progression of adolescents’ mental health. From the standardized indirect effect, the indirect and direct influence coefficient of “family background” on mental health of adolescents is 0.166 and 0.210, respectively. The indirect influence coefficient of partner effect on mental health through school atmosphere is 0.038. This is because peers and good friends are only a minor presence in adolescents’ school life and thus have a smaller impact on their mental health.

### Results analysis of psychotherapy system based on virtual reality

**FIGURE 11:**
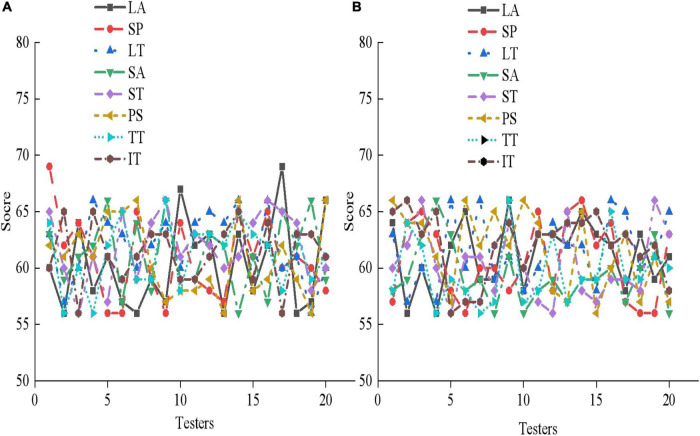
Pre-experiment mental health assessment of 40 adolescent subjects. **(A)** The experimental group; **(B)** the control group.

According to the survey results of mental health of adolescents, 40 adolescents with mental health problems are experimentally studied using the designed mental health therapy system and the VR-based mental health therapy system experiment designed in section “Basic theory of psychology and feasibility analysis of virtual reality.” The rating system of mental health is divided into three grades. The score between 1 and 55 is normal, the score between 56 and 64 is poor or problematic, and the score above 65 is serious. The evaluation criteria include LA, SP, LT, SA, ST, PS, TT, and IT. The results are shown in Figure 11.

**FIGURE 12:**
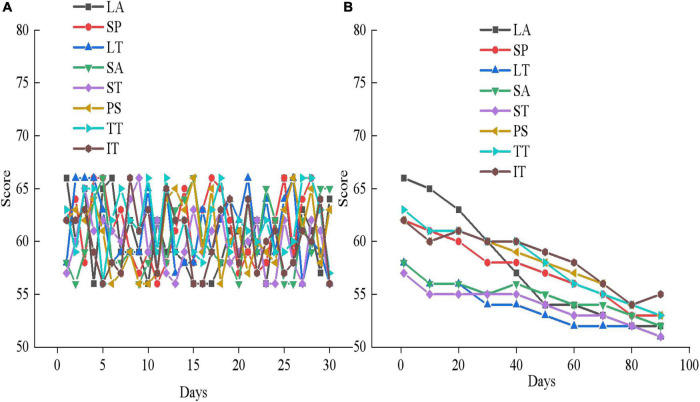
Mental health outcomes of the experimental group in the first month of therapy. **(A)** First month mental health assessment results; **(B)** the mental health changes in experimental group member 1.

**FIGURE 13:**
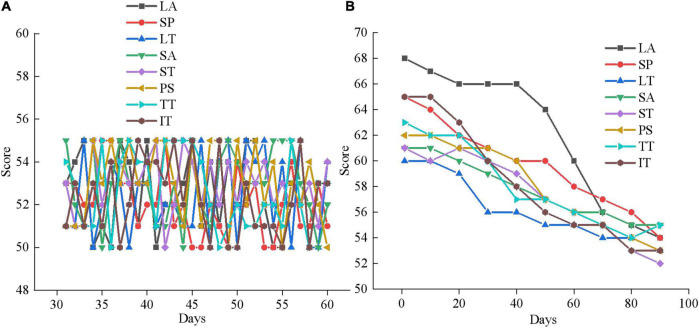
Mental health outcomes of the experimental group in the second month of therapy. **(A)** Second month mental health assessment results; **(B)** the mental health changes of experimental group member 5.

**FIGURE 14:**
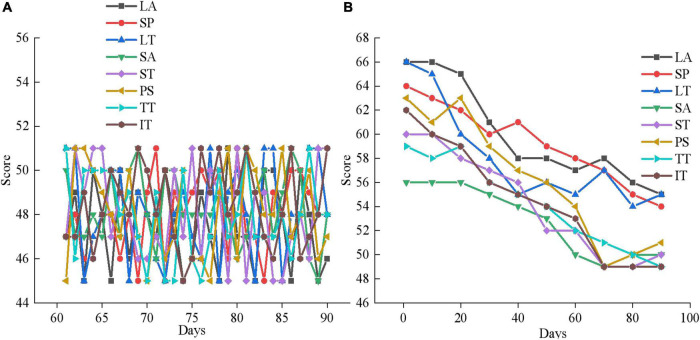
Mental health outcomes of the experimental group in the third month of therapy. **(A)** Third month mental health assessment results; **(B)** The mental health changes of experimental group member 10.

From Figure 11, 20 subjects in the experimental group and 20 subjects in the control group score between 56 and 64 in LA, SP, LT, SA, ST, PS, TT, and IT. Some subjects even score more than 65, indicating that the adolescents that are selected all suffer from serious mental health problems. Then, the psychological therapy system designed in this work is utilized for a mental health therapy for 40 subjects for 3 months, and the results are shown in Figures 12–14.

**FIGURE 15:**
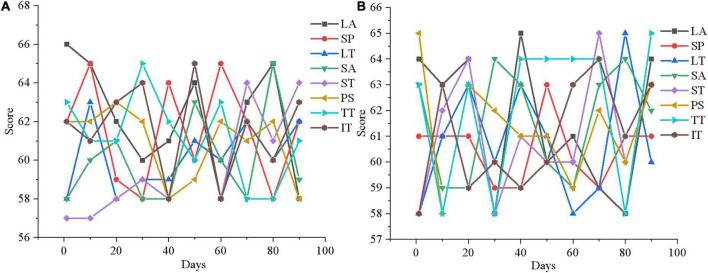
Mental health evaluation results of the control group after 3 months. **(A)** Control group member 1; **(B)** control group member 6.

**FIGURE 16:**
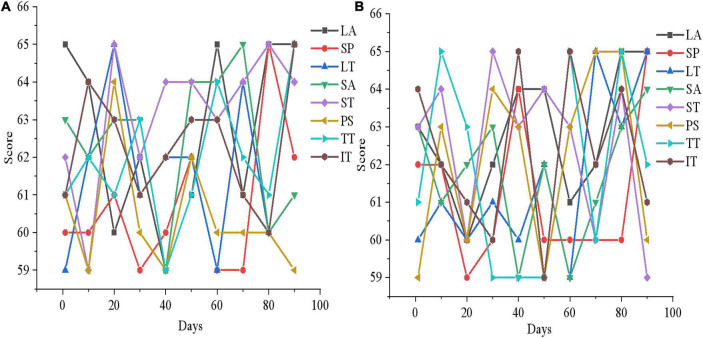
Mental health evaluation results of the control group after 3 months. **(A)** Control group member 10; **(B)** Control group member 12.

**FIGURE 17:**
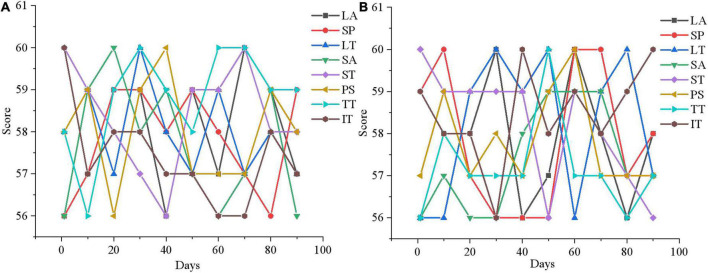
Mental health evaluation results of the control group after 3 months. **(A)** Control group member 15; **(B)** control group member 20.

Figures 12A, 13A, 14A are the average mental health scores of all experimental subjects. Comparison shows that 20 subjects score 56–66 in LA, SP, LT, SA, ST, PS, TT, and IT during the first month of psychotherapy. The score in the second month (50–55 points) is significantly lower than the average of the first month, except for a few exceeding 55. The psychological therapy system designed in this work has obvious therapeutic effects. In Figure 14A in the third month, the scores are significantly lower than those in the second month, with the scores ranging from 45 to 51. It shows that the therapeutic effect of psychotherapy system is relatively stable. In addition, Figures 12B, 13B, 14B show that three subjects are randomly selected, and their mental health problems are significantly alleviated with the help of the psychological therapy system. It shows that the psychological therapy system designed in this work shows excellent therapeutic effect. Then, 6 subjects in the control group are randomly selected and their mental health scores in 3 months are counted, as presented in Figures 15–17.

**FIGURE 18:**
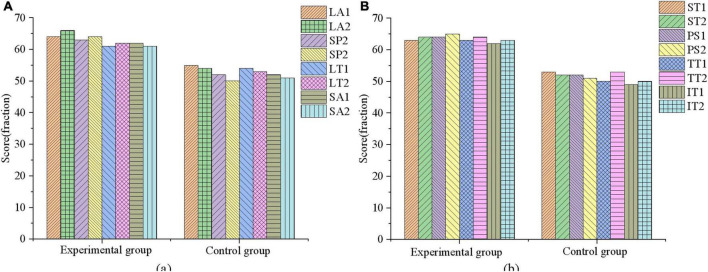
Comparison of mental health evaluation results between experimental group and control group. **(A)** LA, SP, LT, and SA; **(B)** ST, PS, TT, and IT.

Figures 15–17 show that the mental health status of adolescents in the control group is not improved after 3 months, which reflects the effectiveness of the mental health therapy system designed in this work. Then, an experimenter is randomly selected from each of the two groups and the mental health situation is compared 3 months later, as presented in Figure 18.

Figure 18 demonstrates after 3 months of mental health therapy, there is a significant difference between the control group and the experimental group. The mental health status of adolescents in the experimental group is significantly improved, while that in the control group is not, which indicates that the mental health therapy system designed in this work can effectively help adolescents improve their mental health.

## Conclusion

With the development of education industry, multi-directional comprehensive teaching method plays an important role in improving the educational achievement of teenagers. In this work, VR technology is adopted to analyze the influencing factors of adolescent mental health through scientific education. First, based on the application principle of VR technology, the factors that affect adolescent mental health are investigated, and the adolescent psychological treatment system based on VR is designed. Then, under the design of VR technology, the psychological impact of the system is tested experimentally through the practical application and monitoring of the system. Finally, the effect of mental health education under VR technology is evaluated by questionnaire survey. The conclusion is as follows. First, the survey results show that 80% of teenagers are “relatively healthy” or above in terms of mental health. Second, adolescents’ mental health varies significantly in terms of gender, age, only child and non-only child, and whether they have parents or not. Third, after 3 months of psychological treatment, the psychological evaluation scores of 20 subjects in the experimental group are 50–55 points, whereas those of the control group are still 56–65 points. In conclusion, the designed mental health treatment system can effectively help teenagers improve their mental health. However, this work also has some shortcomings. The experimental design of the psychotherapy system is only set up for 3 months, and there are no further studies on whether the treatment effects of the system have long-term effects on the subjects. Therefore, the persistence of the therapeutic effects of the psychotherapy system is unclear. In the future, the research on this aspect will be strengthened to comprehensively promote the development of adolescent psychological education.

## Data availability statement

The original contributions presented in this study are included in the article/supplementary material, further inquiries can be directed to the corresponding author/s.

## Author contributions

All authors listed have made a substantial, direct, and intellectual contribution to the work, and approved it for publication.
